# New insights into ATR inhibition in muscle invasive bladder cancer: The role of apolipoprotein B mRNA editing catalytic subunit 3B

**DOI:** 10.32604/or.2024.048919

**Published:** 2024-05-23

**Authors:** HYUNHO KIM, UIJU CHO, SOOK HEE HONG, HYUNG SOON PARK, IN-HO KIM, HO JUNG AN, BYOUNG YONG SHIM, JIN HYOUNG KANG

**Affiliations:** 1 Division of Medical Oncology, Department of Internal Medicine, St. Vincent’s Hospital, College of Medicine, The Catholic University of Korea, Suwon, Korea; 2 Department of Pathology, St. Vincent’s Hospital, College of Medicine, The Catholic University of Korea, Suwon, Korea; 3 Division of Medical Oncology, Department of Internal Medicine, Seoul St. Mary’s Hospital, College of Medicine, The Catholic University of Korea, Seoul, Korea

**Keywords:** Apolipoprotein B mRNA editing catalytic polypeptide (APOBEC), Ataxia telangiectasia and Rad3-related (ATR), Bladder cancer, DNA damage response, DNA replication stress

## Abstract

**Background:**

Apolipoprotein B mRNA editing catalytic polypeptide (APOBEC), an endogenous mutator, induces DNA damage and activates the ataxia telangiectasia and Rad3-related (ATR)-checkpoint kinase 1 (Chk1) pathway. Although cisplatin-based therapy is the mainstay for muscle-invasive bladder cancer (MIBC), it has a poor survival rate. Therefore, this study aimed to evaluate the efficacy of an ATR inhibitor combined with cisplatin in the treatment of APOBEC catalytic subunit 3B (APOBEC3B) expressing MIBC.

**Methods:**

Immunohistochemical staining was performed to analyze an association between APOBEC3B and ATR in patients with MIBC. The APOBEC3B expression in MIBC cell lines was assessed using real-time polymerase chain reaction and western blot analysis. Western blot analysis was performed to confirm differences in phosphorylated Chk1 (pChk1) expression according to the APOBEC3B expression. Cell viability and apoptosis analyses were performed to examine the anti-tumor activity of ATR inhibitors combined with cisplatin.

**Conclusion:**

There was a significant association between APOBEC3B and ATR expression in the tumor tissues obtained from patients with MIBC. Cells with higher APOBEC3B expression showed higher pChk1 expression than cells expressing low APOBEC3B levels. Combination treatment of ATR inhibitor and cisplatin inhibited cell growth in MIBC cells with a higher APOBEC3B expression. Compared to cisplatin single treatment, combination treatment induced more apoptotic cell death in the cells with higher APOBEC3B expression. **Conclusion:** Our study shows that APOBEC3B’s higher expression status can enhance the sensitivity of MIBC to cisplatin upon ATR inhibition. This result provides new insight into appropriate patient selection for the effective application of ATR inhibitors in MIBC.

## Introduction

Currently, cisplatin-based chemotherapy is the standard chemotherapy regimen for patients with advanced muscle-invasive bladder cancer (MIBC) [1–3]. However, even immune checkpoint inhibitors, as the palliative first-line setting, combined with cisplatin do not improve survival [4–6]. Moreover, although avelumab is approved to be administered as a maintenance treatment following platinum-based chemotherapy, the survival benefit is approximately six months, while a long-term survival benefit is observed in only a limited number of patients [7–9]. Therefore, novel treatment options are required to overcome these limitations and improve the cisplatin efficacy for the treatment of MIBC.

Ataxia telangiectasia and Rad3-related (ATR) kinase is a key checkpoint molecule that initiates DNA damage response when DNA damage occurs at specific sites within single-stranded DNA, including stressed replication forks [10,11]. Therefore, ATR inhibition can force cell cycle progression with incomplete DNA, inducing cell apoptosis [12]. Previous *in vitro* studies have presented the potential activity of several ATR inhibitors in various cancer cell lines [12–16]. However, a clinical phase 2 study using ATR inhibitors in MIBC failed to show significant survival improvement [17]. Although statistical significance was not achieved, the combination of ATR inhibitor and cisplatin-based chemotherapy showed a numerically improved survival [17]. Moreover, another study on ovarian cancer conducted at the same time succeeded in deriving significant results [18]. Therefore, the study raised a need to select suitable patients with MIBC for ATR inhibitor treatment.

Apolipoprotein B mRNA editing catalytic subunit 3B (APOBEC3B), an endogenous carcinogen, is overexpressed in approximately two-thirds of patients with MIBC [19,20], and its overexpression is associated with enhancement of the ATR signaling pathway [19–22]. This may be because APOBEC3B induces ATR activity by increasing replication stress in the process of cytidine deamination to create an abasic site [22,23]. Similarly, a previous cell line study also reported a relationship between APOBEC3B overexpression and anti-cancer activity of ATR inhibition [22].

Accordingly, we hypothesized that the combination of ATR inhibitors and cisplatin in APOBEC3B-overexpressing MIBC may be more effective in increasing the sensitivity of MIBC to cisplatin. Therefore, our study aimed to evaluate the potential of a combination treatment strategy of cisplatin and ATR inhibitor in APOBEC3B-high expressing MIBC.

## Materials and Methods

### Patient population and tissue samples

This study evaluated patients diagnosed with bladder cancer between 2013 and 2021 at St. Vincent’s Hospital, Suwon, Republic of Korea. The study was approved by the Institutional Review Board of St. Vincent’s Hospital of the Catholic University of Korea (grant numbers: VC21ZASI0036 and VC20SISI0187) and was conducted in accordance with the principles of the Declaration of Helsinki and its later amendments. Written informed consent was obtained from all participants.

All patients underwent surgery, including transurethral resection of their bladder tumor or radical cystectomy. The pathological diagnosis was muscle-invasive urothelial carcinoma in all included patients. A formalin-fixed, paraffin-embedded tumor block was used to evaluate APOBEC3B protein expression, and patient medical records were thoroughly reviewed. All clinical information was extracted anonymously in a de-identified manner.

### Immunohistochemistry to interpret APOBEC3B expression

Immunohistochemistry (IHC) staining was performed as described previously [24]. Rabbit polyclonal anti-APOBEC3B antibody (ab191695, Abcam, Cambridge, UK, dilution 1:200) was used as a primary antibody against human APOBEC3B, and rabbit monoclonal anti-ATR (phospho S428) antibody (ab178407; Abcam, Cambridge, UK, dilution 1:200) was used as a primary antibody against human ATR. The nuclear and cytoplasmic staining of APOBEC3B and ATR in each sample was evaluated by an independent pathologist using a semiquantitative score. The staining intensity was interpreted as 2+ of the control, 1+ of weaker, 3+ of stronger, and 0 of negative staining. The H-scores were calculated on a scale of 0–300 by multiplying the staining intensity (0, no staining; 1, weak; 2, moderate; and 3, strong) according to the percentage of cells (0%–100%) at each intensity level.

### Cell lines and culture conditions

The MIBC cell lines evaluated in this study (HT-1376, 5637, HT1197, and 253 J) were obtained from the Korean Cell Line Bank (KCLB; Seoul, Republic of Korea). The cell lines were cultivated according to KCLB recommendations. Briefly, the cells were cultured in Dulbecco’s Modified Eagle Medium (DMEM; 90%) with fetal bovine serum (FBS; 10%), penicillin, and streptomycin at 37°C in a humidified environment.

### Real-time polymerase chain reaction analysis

Real-time polymerase chain reaction (PCR) was performed to confirm the expression of APOBEC3B messenger RNA (mRNA) in the cell lines. RNA was extracted from each cell using an RNA-spin^TM^ Total RNA Extraction Kit (iNtRON Biotechnology, Seoul, Republic of Korea). The extracted RNA was quantified using a NanoDrop^TM^ Spectrophotometer (Thermo Fisher Scientific, Waltham, MA, USA), and 2 µg of complementary DNA (cDNA) was subsequently synthesized using an AMPEGENE^®^ cDNA Synthesis Kit (Enzo Life Sciences, Farmingdale, NY, USA). Real-time PCR was performed using DNA Master SYBR Green I and Light Cycler 2.0 real-time PCR equipment (Roche, Basel, Switzerland). The primers used for human APOBEC3B were GACCCTTTGGTCCTTCGAC (sense) and GCACAGCCCCAGGAGAAG (antisense). The primers for human β-actin were GTCCACCTTCCAGCAGATGT (sense) and AAAGCCATGCCAATCTCATC (antisense). The detailed experiments were performed as previously described [21].

### Western blot analysis

Protein extraction from each sample was performed using radioimmunoprecipitation assay lysis and extraction buffer (Thermo Fisher Scientific, Waltham, MA, USA). The protein levels were quantified using the Bradford assay (Thermo Fisher Scientific, Waltham, MA, USA). For each evaluation, 2 µg of protein was transferred to a polyvinylidene difluoride membrane following sodium dodecyl sulfate-polyacrylamide gel electrophoresis. The transferred membranes were blocked with 5% non-fat milk, and each primary antibody was incubated overnight at 4°C. Horseradish peroxidase-conjugated secondary antibodies were incubated for 1 h. Images were captured using an ImageQuantTM LAS4000 camera system (GE Healthcare, Chicago, IL, USA). The antibodies used in our study were APOBEC3B (ab191695; Abcam, Cambridge, UK), Chk1 (sc-8408; Santa Cruz Biotechnology, Dallas, TX, USA), phospho-Chk1 (Ser317, #2344; Cell Signaling Technology, Danvers, MA, USA), H2AX (#2595, Cell Signaling Technology, Danvers, MA, USA), phospho-H2AX (Ser139, #9718; Cell Signaling Technology, Danvers, MA, USA), and β-actin (#4967, Cell Signaling Technology, Danvers, MA, USA). The ATR inhibitor used in this study was VE-821 (Selleckchem, Houston, TX, USA).

### Cell viability assays

Each cell line was seeded in a 96-well plate (5 × 103 in 100 µL), and reagents were added depending on concentrations and timings within various assays, as described below. Cell viability was determined using a Cell Counting Kit (CCK; EZ-Cytox Cell-Based Assay, DoGenBio Co., Ltd., Seoul, Korea). According to the CCK Assay Kit protocol, 10 µL of CCK was added to each plate, and the cell count was read at 490 nm using a microplate reader 1–4 h after incubation; cell viability was measured 48 h after drug treatment, depending on various drug concentrations. In the time-dependent assay, cell viability was evaluated using 3 µM cisplatin and 2.5 µM VE-821, an ATP competitive ATR inhibitor, and incubation was maintained for up to 72 h.

### Assessment of cell apoptosis and cell cycle analysis

Annexin V and propidium iodide (PI) apoptosis assay kits (Thermo Fisher Scientific, Waltham, MA, USA) were used according to the manufacturer’s instructions. Cells treated with the apoptosis assay kit were evaluated using flow cytometry. The detailed experimental process was described previously [20]. Annexin V-negative and PI-negative cells were considered viable, annexin V-positive and PI-negative cells were considered early apoptotic cells, and annexin V-positive and PI-positive cells were considered late apoptotic cells. For cell cycle analysis, samples were stained using a BD Cycletest_Plus DNA Kit and assessed using flow cytometry by Beckman Coulter’s Navios model. The result was analyzed using Beckman Coulter’s kaluza analysis version 2.1 program.

### Statistical analysis

The differences in APOBEC3B expression and cell viability in each cell line were compared using *t*-test and Mann–Whitney U test. APOBEC3B expression levels and response rates to chemotherapy were compared using chi-square tests. The association between APOBEC3B and ATR was also analyzed using a chi-square test. All statistical analyses were performed using SPSS statistical software version 25 (IBM Corp., Armonk, NY, USA). Results with a two-sided *p* < 0.05 were considered statistically significant.

## Results

### APOBEC3B expression and tumor response to cisplatin in patients with MIBC

**Figure 1:**
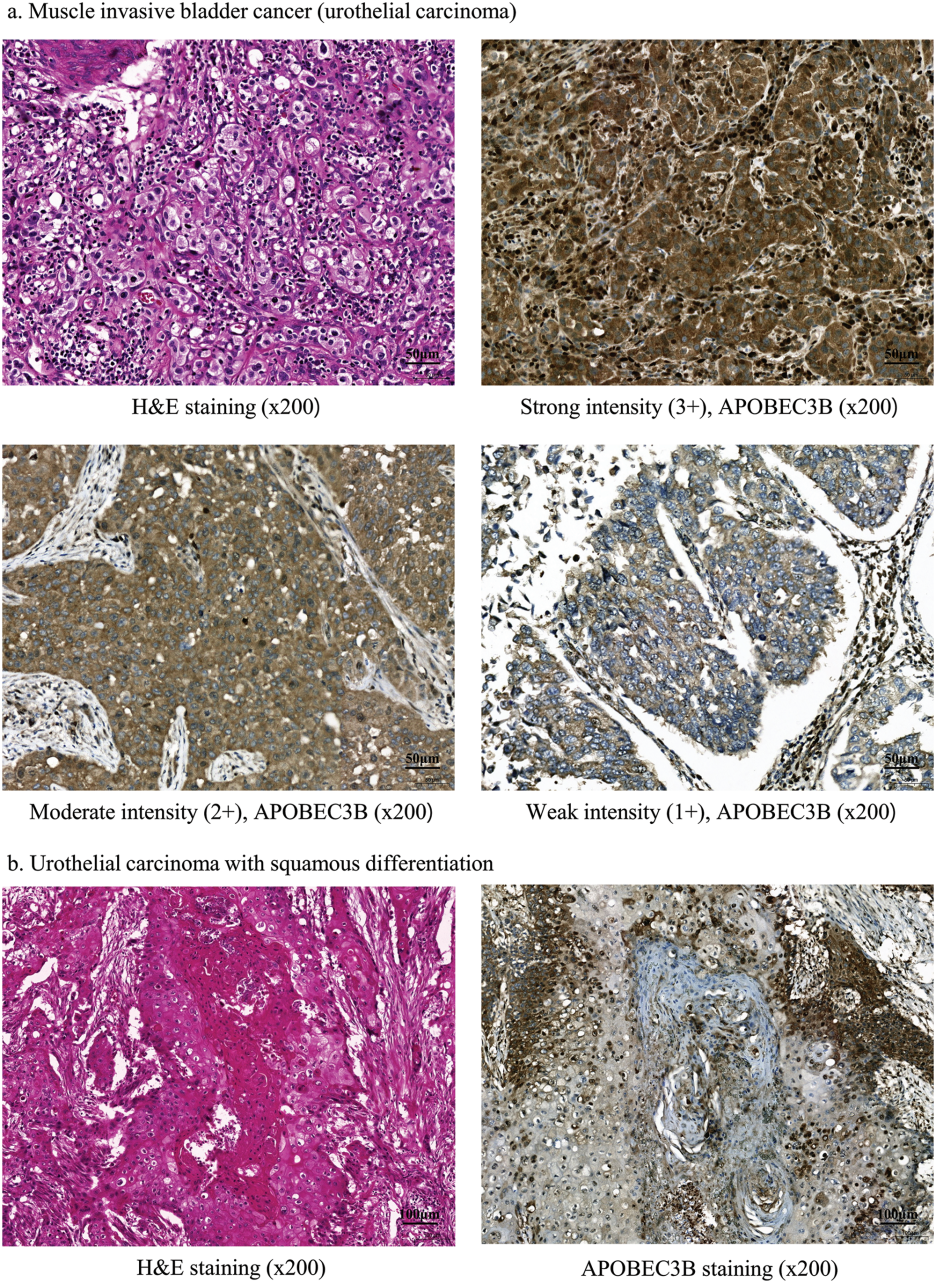
Apolipoprotein B mRNA editing enzyme catalytic subunit 3B (APOBEC3B) expression following immunohistochemical staining in patients with bladder cancer. (a) Findings for muscle invasive bladder cancer (MIBC; urothelial carcinoma). Tissue samples from patients with bladder cancer immunohistochemically stained for APOBEC3B expression (ab191695; Abcam, Cambridge, UK, dilution 1:200). (b) Squamous differentiation. There is almost no staining with the APOBEC3B antibody in the squamous differentiated area.

We first evaluated APOBEC3B expression using immunohistochemical staining in samples obtained from 61 patients with MIBC (Suppl. Table S1). Strong (3+), moderate (2+), and weak (1+) intensities of APOBEC3B expression were observed in 43 (70.5%), 16 (26.2%), and 2 (3.3%) patients, respectively (Fig. 1a). No instances of negative APOBEC3B expression were observed. We classified patients into the high (3+) and low expression groups (2+ or less) according to the intensity of APOBEC3B protein expression. The mean H-score significantly varied between the high and low APOBEC3B expression groups (*p* < 0.0001). Notably, the squamous-differentiated area of the tumor was determined to be almost unstained with the APOBEC3B antibody (Fig. 1b). No associations between tumor response and APOBEC3B were observed. Additionally, neither TNM stage nor tumor recurrence was associated with APOBEC3B expression (Suppl. Table S1).

### ATR expression and its association with APOBEC3B in patients with MIBC

**Figure 2:**
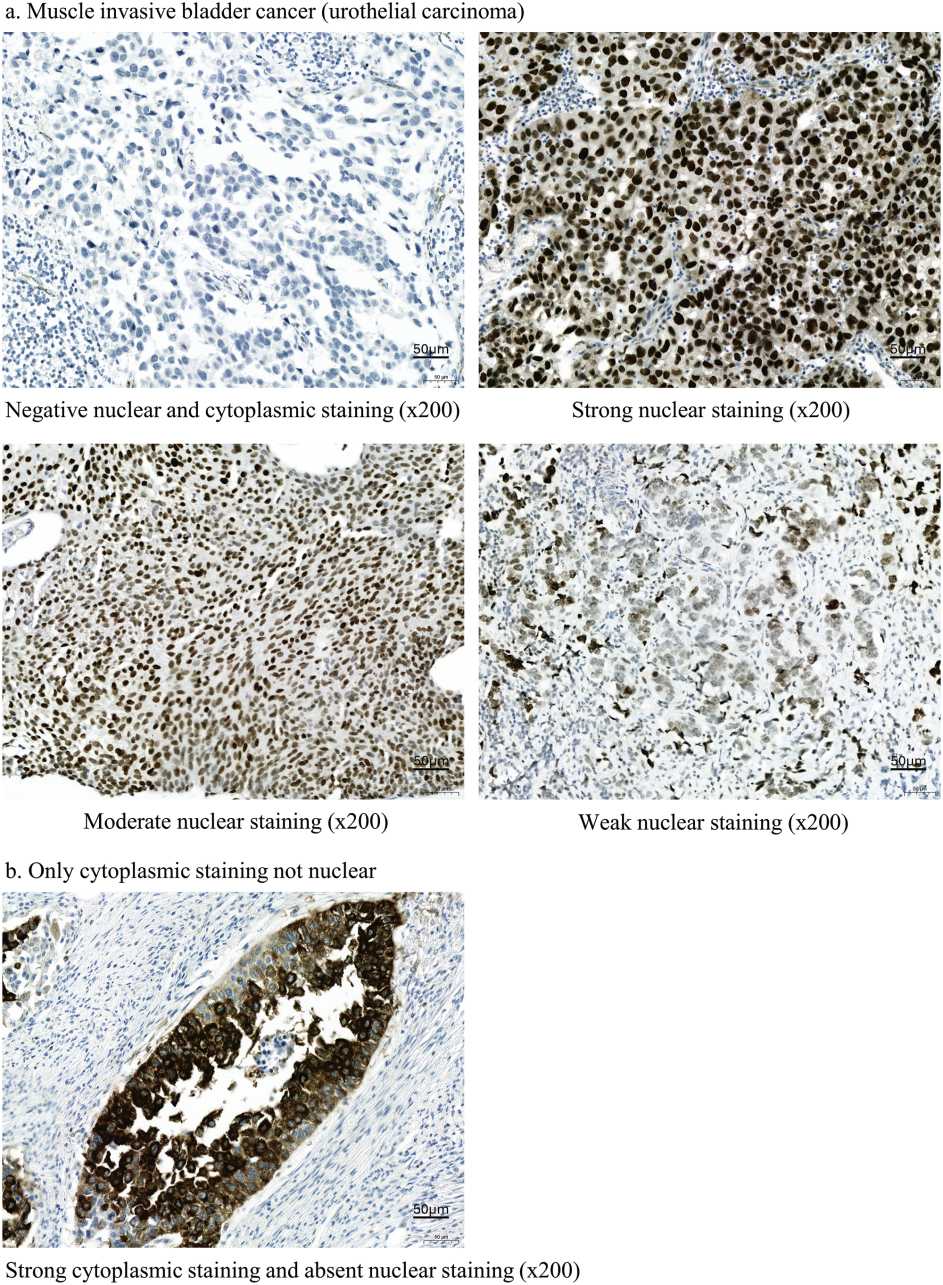
Ataxia telangiectasia and Rad3-related (ATR) kinases expression following immunohistochemical staining in patients with bladder cancer. (a) Findings for muscle invasive bladder cancer (MIBC; urothelial carcinoma). Tissue samples from patients with bladder cancer immunohistochemically stained for ATR expression (ab178407; Abcam, Cambridge, UK). (b) Results for nuclear and cytoplasmic staining. ATR is stained in the cytoplasm but not in the nucleus.

Immunohistochemical staining (Fig. 2a) revealed that similar to APOBEC3B, ATR was expressed in over 50% of patients with MIBC. Strong (3+), moderate (2+), and negative intensity of ATR expression was observed in 36 (59.0%), 5 (8.2%), and 20 (32.8%) patients, respectively. Similar to the interpretation for APOBEC3B, we classified patients into high (3+) and low (2+ or less) expression groups according to ATR expression intensity. Cytoplasmic ATR expression without nuclear staining was interpreted as negative (Fig. 2b). A significant correlation was observed between ATR and APOBEC3B expressions (*p* = 0.039, OR = 3.255) which was linear (*p* = 0.040) (Table 1). As for clinical factors, recurrence was associated with ATR expression. However, response to cisplatin was not associated with ATR expression (Suppl. Table S2).

**Table 1:** The association between Apolipoprotein B mRNA editing enzyme catalytic subunit 3B (APOBEC3B) and Ataxia telangiectasia and Rad3-related (ATR) kinases expression in bladder cancer patients

		ATR expression			
		Low	High	Total	*p*
APOBEC3B expression	Low	11 (61.1%)	7 (38.9%)	18 (100%)	0.039
	High	14 (32.6%)	29 (67.4%)	43 (100%)	

Note: APOBEC3B expression levels are correlated with ATR expression (Chi-square test, *p* = 0.039, odds ratio = 3.255 [95% CI 10.201]). ATR expression increases with APOBEC3B expression (linear by linear association [Chi-square test for trend], *p* = 0.040).

### APOBEC3B expressions and ATR-Chk1 signals in bladder cancer cell lines

**Figure 3:**
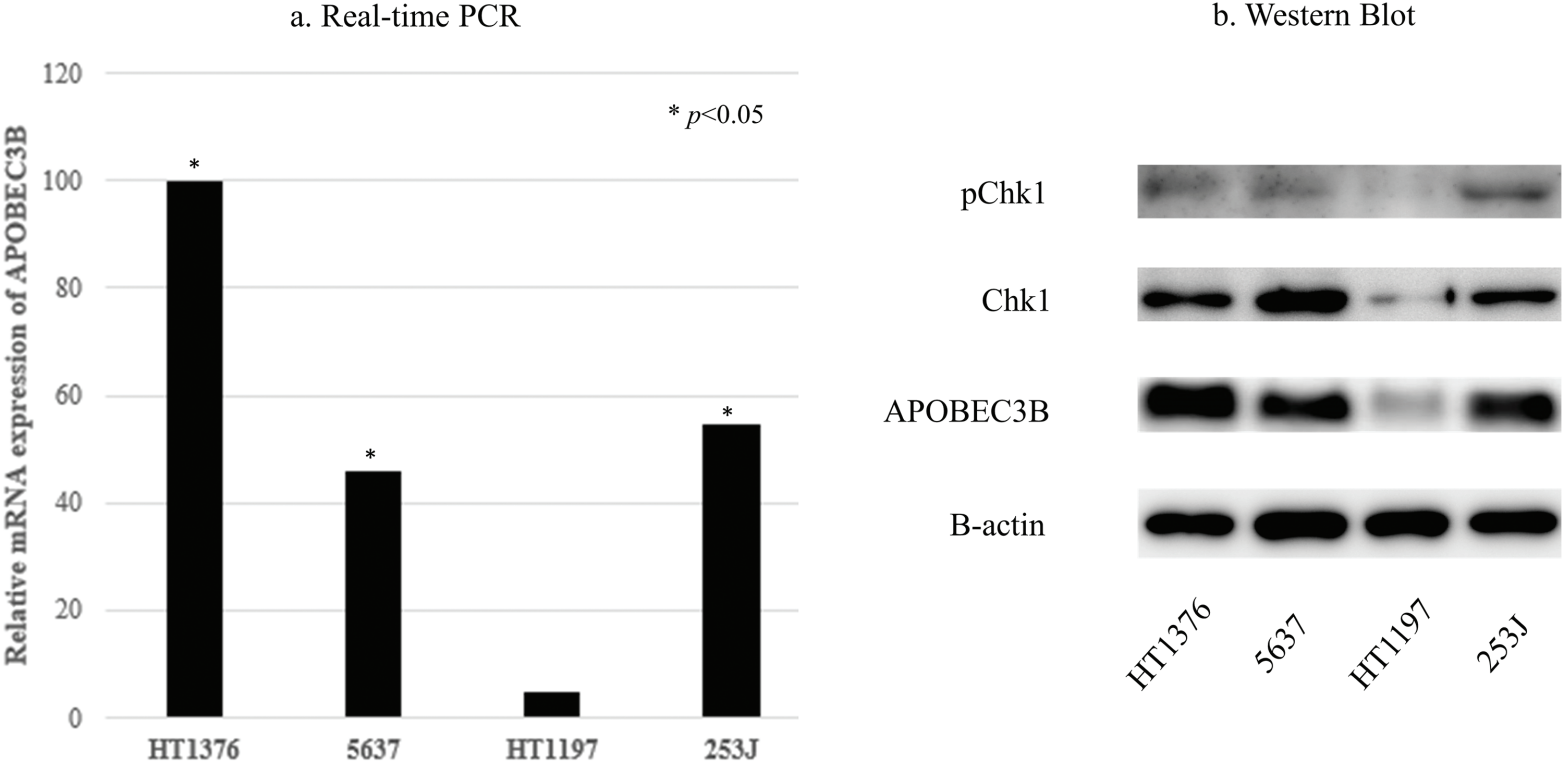
Apolipoprotein B mRNA editing enzyme catalytic subunit 3B (APOBEC3B) expression and ataxia telangiectasia and Rad3-related (ATR) activity in bladder cancer cell lines. (a) Real-time polymerase chain reaction (PCR) findings. APOBEC3B mRNA expression is significantly different between cell lines expressing high levels of APOBEC3B (HT1376, 5637, 253J) and the cell line expressing low levels of APOBEC3B (HT1197; *p < 0.05). Relative values (1/2^−∆∆Ct^) of HT1376, 5637, 253J, and HT1197 are 0.00999, 0.00457, 0.00544, and 0.000494, respectively, using lightcycler software 4.1. (b) Western blotting. APOBEC3B protein expression is consistent with the mRNA expression levels observed in this study. Cell lines expressing high levels of APOBEC3B have more prominent Chk1 expression than the cell lines expressing low levels of APOBEC3B.

As for real-time PCR results, HT-1376, 5637, and 253J MIBC cells showed higher mRNA APOBEC3B expression than HT1197 cells (Fig. 3a). Therefore, we designated HT-1376, 5637, and 253J cell lines as the cells with higher expression of APOBEC3B and the HT-1197 cell line as the cells with lower expression of APOBEC3B.

The APOBEC3B protein expression levels were consistent with the mRNA expression levels in MIBC cell lines (Fig. 3b). The expressions of Chk1 and pChk1 tended to increase in MIBC cells with higher APOBEC3B expression, compared to MIBC cells with lower APOBEC3B expression (Fig. 3b). VE-821 treatment (5 µM) increased γH2AX expression and decreased pChk1 expression, but not Chk1 expression (Suppl. Fig. S1).

### Antitumor activities of ATR inhibitor and cisplatin alone and their combination in bladder cancer cell lines

**Figure 4:**
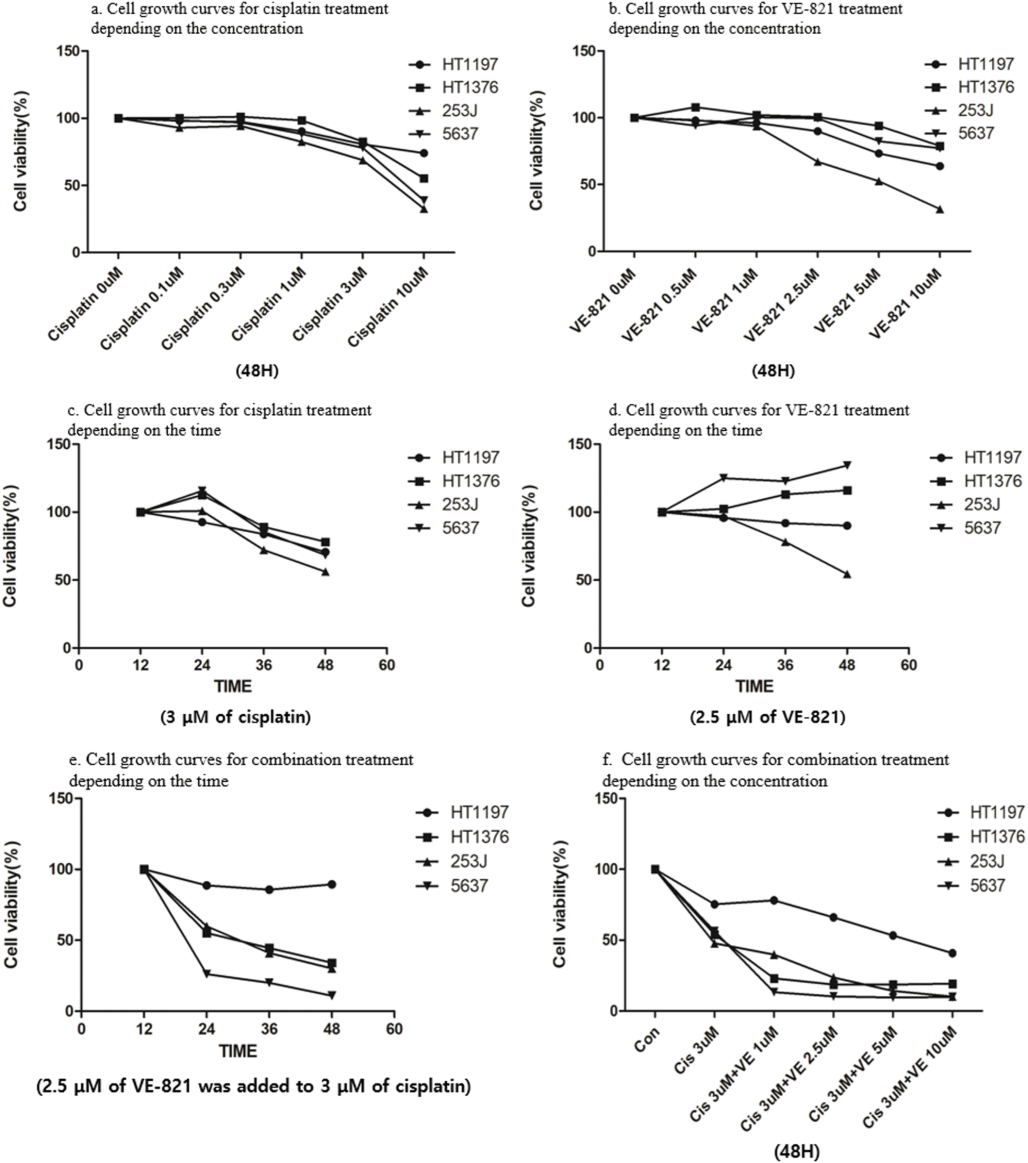
Cell viability assays following treatment with cisplatin and ataxia telangiectasia and Rad3-related (ATR) inhibitor (VE-821) in bladder cancer cell lines. (a) Concentration dependent cell growth curves for cisplatin treatment. Regardless of APOBEC3B expression, cisplatin shows a dose-dependent effect at 48 h in MIBC cells. (b) Concentration dependent cell growth curves for VE-821 treatment. Regardless of APOBEC3B expression, VE-821 shows a dose-dependent effect at 48 h in MIBC cells. (c) Time dependent cell growth curves for cisplatin treatment. A low dose of cisplatin (3 μM) is administered in all cell lines. (d) Time dependent cell growth curves for VE-821 treatment. All cell lines are treated with low doses of VE-821 (2.5 μM). The efficacy of combination therapy (c) is more pronounced than that of low doses of both monotherapies, which were determined to be ineffective. (e) Time dependent cell growth curves for combination treatment: 2.5 μM of VE-821 was added to 3 μM of cisplatin in all cell lines. Combination therapy shows a synergistic effect in high-expressing APOBEC3B cell lines compared to that in the low-expressing APOBEC3B cell line. (f) Concentration dependent cell growth curves for combination treatment. The addition of VE-821 is effective even at a low concentration (1 μM) in the high-expressing APOBEC3B cell lines.

Cisplatin alone inhibited cell proliferation in a dose-dependent manner in MIBC cells with higher APOBEC3B expression. Additionally, low cisplatin (3 µM) modestly inhibited cell proliferation at 48 h. Whereas VE-821 alone showed anti-proliferative activity at lower concentration (2.5 µM) only for 253J cells at 48 h, but not in other MIBC cells (Figs. 4a, 4b). For dose determination of the combination, the cisplatin and VE-821 doses were fixed, and cell growth curves were observed over time. Low-dose cisplatin (3 µM) modestly inhibited cell growth regardless of cell lines. Whereas low dose VE-821 (2.5 µM) did not show any antitumor activity over time in cell viability assays (Figs. 4c–4d).

To enhance their antitumor effect, we implemented a combination treatment. Considering the possibility of synergistic interaction between two drugs, low doses (3 µM for cisplatin and 2.5 µM for VE-821), which showed insufficient effect with a single treatment, were selected as the combination concentration. VE-821 (2.5 µM) in combination with cisplatin (3 µM) greatly suppressed cell viability in MIBC cells expressing higher APOBEC3B levels but not in HT1197 cells expressing lower APOBEC3B levels (Fig. 4e). The difference in antitumor activity appeared at 24 h after combination treatment and gradually increased as time elapsed (Fig. 4e).

Notably, the anti-proliferative activity of the combination treatment was pronounced even at the lower concentration of VE-821 (1 µM) in the MIBC cells with higher APOBEC3B expression (Fig. 4f). As the concentration of VE-821 increased, cell growth inhibition gradually increased in HT-1197 cells, which were insensitive to VE-821 (2.5 µM) single treatment. The antitumor activity of combination treatment was more evident in cells expressing higher APOBEC3B when the cell growth curve for a single treatment of two drugs was compared with that of the combination treatment (Figs. 4c–4e).

### Induction of apoptotic cell death by ATR inhibition and cisplatin

**Figure 5:**
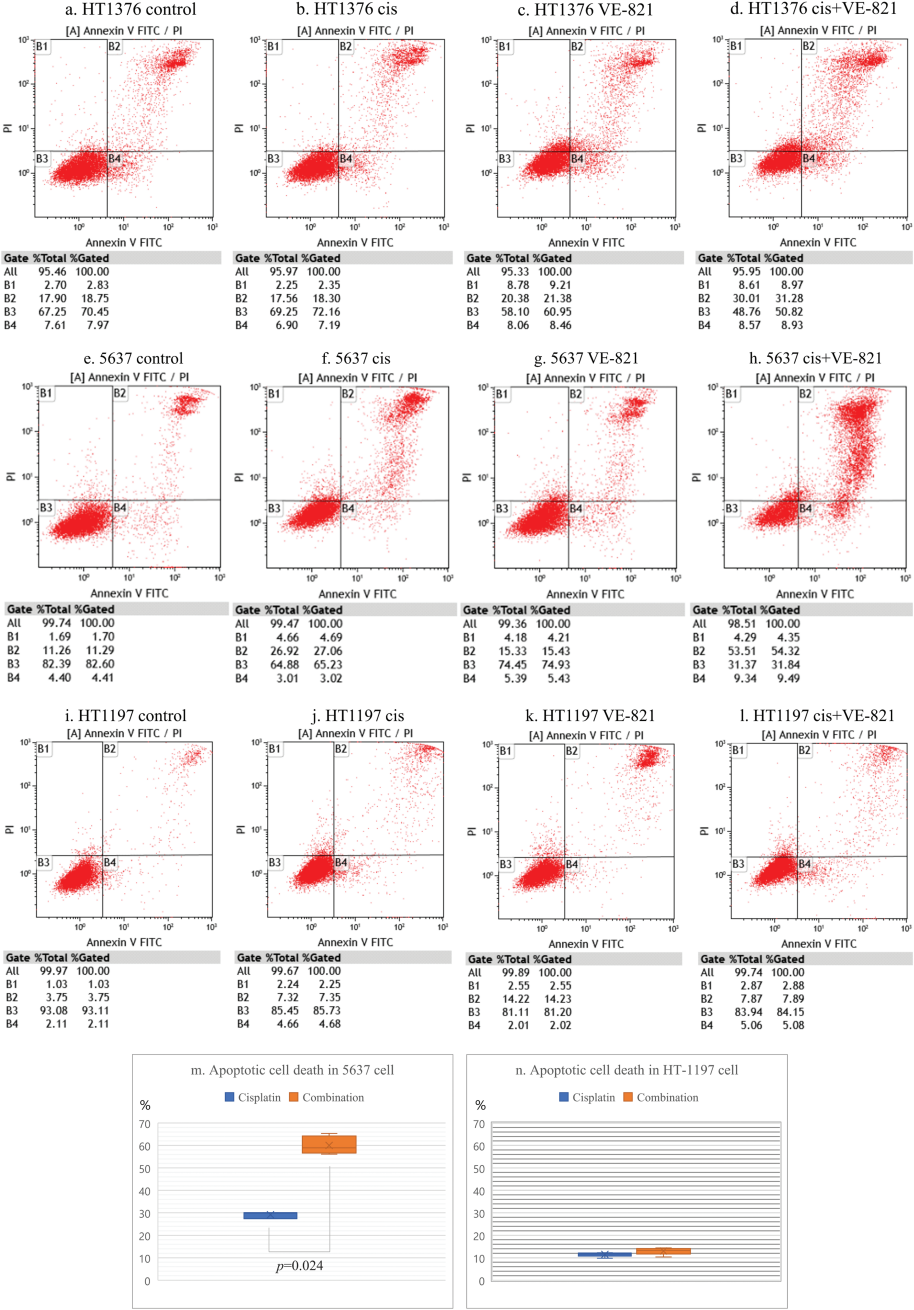
Apoptotic cell death by ataxia telangiectasia and Rad3-related (ATR) inhibition in bladder cancer cells showing high expression of apolipoprotein B mRNA editing enzyme catalytic subunit 3B (APOBEC3B). (a) Findings in comparison with the control group. The control group comprised untreated HT-1376 cells. Flow cytometry was performed to identify apoptotic cell death. (b) Results of cisplatin single treatment. There is almost no induction of apoptotic cell death in HT-1376 cells treated with low-dose cisplatin (1 µM). (c) Findings in VE-821 (an ATR inhibitor) single treatment. VE-821 shows little induction of apoptotic cell death in HT-1376 cells treated with low dose VE-821 (2.5 µM). (d) Results for evaluations of the combination treatment in HT-1376 cells. The combination of cisplatin (1 µM) and VE-821 (2.5 µM) induces apoptotic cell death at 48 h after treatment. (e–h) Findings in 5637 cells. Flow cytometry was performed to identify apoptotic cell death. (i–l) Results of HT-1197 cells. Flow cytometry was performed to identify apoptotic cell death. (m, n) Comparison apoptotic cell deaths between cisplatin single treatment and combination treatment in 5637 and HT-1197 cells.

We performed flow cytometric analysis of cell apoptosis induced by the combination treatment lower concentrations of cisplatin (1 µM) and VE-821 (2.5 µM). Both VE-821 and cisplatin alone had little induction of apoptotic cell death in HT-1376, 5637, and HT-1197 cells, whereas their combination induced apoptotic cell death in both HT-1376 and 5637 cells with higher APOBEC3B expression. Notably, apoptosis induced by the combination treatment was most pronounced in 5637 cells. Compared with cisplatin alone, apoptotic cell death by the combination treatment in the 5637 cells significantly increased from 30.1% to 63.8% (Figs. 5a–5h). In contrast, no additional apoptosis was found in HT-1197 cells with lower APOBEC3B expression (Figs. 5i–5n).

### Cell cycle progression by ATR inhibition and cisplatin

The cell cycle states impacted by the combination treatment (1 µM for cisplatin and 2.5 µM for VE-821) were different depending on the APOBEC3B expression. No significant cell cycle changes occurred in the 5637 cells treated with VE-821 alone. However, when cisplatin alone was administered, the G0-G1 subpopulation decreased, and the S subpopulation notably increased. Meanwhile, VE-821 combined with cisplatin markedly reduced the G2-M subpopulation and greatly increased the S phase subpopulation compared to cisplatin alone (Suppl. Fig. S2a). However, no cell cycle changes were observed in HT-1197 with a lower expression of APOBEC3B (Suppl. Fig. S2b).

## Discussion

This study aimed to investigate the differential effects of cisplatin and ATR inhibitor combination treatment based on APOBEC3B expression in patients with MIBC. The combination treatment proposed herein effectively suppressed cell growth when APOBEC3B expression was higher. In contrast, no difference was observed when APOBEC3B expression was lower. These results indicate that ATR inhibitors can increase the sensitivity to cisplatin in patients with MIBC with higher APOBEC3B expression. Thus, it is crucial to identify patients with MIBC suitable for clinical treatment using ATR inhibitors.

The most well-known epidemiologic cause of bladder cancer is smoking [25]. However, genomic analyses reveal that MIBC primarily exhibited in the APOBEC3-mediated mutations and not in smoking-related mutations [19,26], suggesting that APOBEC3B plays an important role in MIBC pathogenesis. Moreover, APOBEC3B overexpression is reportedly observed in approximately two-thirds of patients with MIBC [19,24]. Therefore, a biologically deeper analysis and understanding of APOBEC3B overexpression is necessary for developing treatment strategies. Similarly, our study observed APOBEC3B overexpression in approximately 70% of patients with MIBC.

We observed a significant correlation between APOBEC3B and ATR expression in patient tissue samples and MIBC cells. This result is consistent with that of previous studies [12,22]. In terms of the correlation, Buisson et al. showed that APOBEC3B high expression induced abasic sites and increased stress at the replication fork, leading to ATR pathway activation to protect cancer cells from replication stress [22]. Swanton et al. presented that the expression of pRPA (S33; a marker of replication stress) decreased in APOBEC3B knockdown lung cancer cells compared to APOBEC3B-expressing lung cancer cells [23]. We also observed that ATR inhibition induces DNA damage in APOBEC3B high expression cells by analyzing molecules such as pChk1 or γH2AX. To elaborate in detail, APOBEC3B catalyzes the deamination of cytosine to uracil in DNA, and the substituted uracil is subsequently removed by uracil DNA glycosylase, creating abasic sites [27]. This induced DNA damage leads to replication stalling and increased replication stress [22]. The exposed single-stranded DNA at the slowed replication fork is bound by RPA, which recruits ATRIP, Rad17, TopBP1, and others [11]. This complex activates ATR, which in turn phosphorylates CHK1 [11]. This process stabilizes the replication fork and regulates checkpoints, allowing replication to complete [11]. Therefore, ATR inhibition results in checkpoint defects and a replication catastrophe, suppressing cell growth and survival. This suggests ATR as a novel therapeutic target in MIBC with high levels of APOBEC3B expression.

The assumption that ATR inhibition would be effective in a state of increased replication stress can also be inferred from a previous clinical trial, wherein patients with ovarian cancer showed prolonged survival with ATR inhibition plus DNA damaging cytotoxic chemotherapy [18]. Conventionally, ovarian cancer has increased replication stress [28]. Ovarian cancer harbors a loss of cell cycle checkpoints related to *TP53* mutations, premature cell cycle progression due to cyclin E1 (CCNE1) amplification, and deficiencies in DNA repair processes [18]. Similarly, MIBC with high levels of APOBEC3 expression exhibits more genetic alterations related to the cell cycle checkpoint, including *TP53* and *ATM*, compared to MIBC expressing low levels of APOBEC3 [19]. Cisplatin reportedly acts as a DNA adductor via cross-linking between nucleotide bases [29]. This crosslink slows the progression of the cell cycle and induces abnormal cell cycle arrest through the activation of the Chk1 pathway [29,30]. Therefore, if ATR inhibition is applied in MIBC with high APOBEC3B expression, which occurs in a state of increased replication stress, the DNA damage induced by cisplatin will be more difficult to repair than usual, resulting in a greater increase in abnormal cell cycle arrest and apoptosis compared to cisplatin alone. We analyzed this using MIBC cells with varying expressions of APOBEC3B. As expected, the combination treatment in higher APOBEC3B MIBC increased apoptosis compared to cisplatin alone. Further, in cell cycle analysis, we observed a decrease in the proportion of cells in the G0-G1 and G2 phases and an increase in the S and M phases, suggesting that the changes may have led to decreased preparation phase and increased active phase in the cell division cycle, ultimately contributing to the increased cisplatin sensitivity.

Taken together, patients with MIBC with a higher APOBEC3B expression may be an effective target population for cisplatin-based chemotherapy and ATR inhibitor combination treatment.

However, our study has several limitations. First, our results reporting a relationship between ATR and APOBEC3B must be interpreted cautiously due to the small sample size. Referring to previous studies using public data from The Cancer Genome Atlas and the Beijing Genomics Institute, APOBEC3 in MIBC has been shown to be related to DNA damage genes, including ATR [19]. More extensive research in diverse cohorts with larger sample sizes is needed for validation.

Additionally, our study’s sample size was inadequate to analyzing clinical characteristics associated with APOBEC3B expression in MIBC. In our previous study, differences in survival were observed in patients with metastatic bladder cancer based on APOBEC3B expression, and a potential relation with tumor-infiltrating lymphocytes was proposed [24]. Another study has also shown that bladder cancer with high APOBEC3B expression has a better prognosis compared to those with low expression, and higher infiltration of various immune cells and expression of immune checkpoints, including CD276 [31]. Thus, it is necessary to analyze the possibility of a correlation between APOBEC3B and basic clinical information in various cohorts.

Next, our study did not conduct a detailed molecular evaluation of how ATR inhibition increases cisplatin sensitivity. This analysis could have strengthened the mechanistic understanding of why ATR inhibition was effective only in cases of high APOBEC3B expression observed in our study. However, Sigala et al. previously reported that ATR is involved in the translocation of Serine-arginine protein kinases related to basic cellular processes such as mRNA splicing, chromatin reorganization, and cell cycle regulation into the nucleus, which could be linked to the DNA damage pathway and influence sensitivity to chemotherapeutic agents [32].

Lastly, we did not create APOBEC3B overexpressed or silenced cells using methods like siRNA to investigate the function of APOBEC3B under *in vitro* conditions. This is a notable limitation, as cell lines with modulated APOBEC3B expression could unveil deeper mechanisms linking ATR inhibitors and APOBEC3B expression, alongside showcasing their clinical relavance. To address this concern, our study was conducted using cell lines with different levels of APOBEC3B expression. Considering the rarity of negative APOBEC3B expression in MIBC, we believe that our approach is likely to offer insights with clinical implications. As replication stress is influenced by a variety of factors [30], there could be several uncontrolled variables affecting our results besides APOBEC3B expression. Additionally, we did not explore the potential effects of the relationship between APOBEC3B and other APOBEC3 family members. Recent reports suggest that APOBEC3A and APOBEC3B can influence each other’s expression and activity in relation to APOBEC mediated mutation burden [33], indicating the need for further research in this area.

Therefore, caution is necessary when interpreting our results, and further investigations are warranted to explore not only the ATR-Chk1 pathway but also various factors that can increase replication stress [30]. Our findings should be considered as highlighting the need for additional research into one of the potential options that could enhance the efficacy of existing standard treatments for MIBC.

In conclusion, our study demonstrated that a higher expression of APOBEC3B enhances the sensitivity of MIBC to cisplatin upon ATR inhibition. This is associated with the upregulation of the ATR-Chk1 pathway and induction of DNA damage and immature cell cycle progression upon ATR inhibition. These findings explain why the combination of cisplatin and ATR inhibitors in MIBC with higher expression of APOBEC3B led to a more pronounced inhibition of cell growth and increased apoptotic cell death compared to cisplatin alone, while no such effects are observed in lower expression of APOBEC3B. To the best of our knowledge, this is the first *in vitro* study on the selective application of an ATR inhibitor in MIBC cell lines. Furthermore, a correlation between APOBEC3B and ATR expression was observed in actual patient tissues, with a high expression of both proteins observed in approximately two-thirds of patients with MIBC. These results provide new information about appropriate patient selection for the effective application of ATR inhibitors in MIBC. Moreover, as the effect of immune checkpoint inhibitors can be closely related to platinum sensitivity [8,34], our study results related to platinum sensitivity not only provide the possibility of enhancing the therapeutic effect of cisplatin in MIBC but also potentially offer translationally important insights into the new treatment strategies

## Supplementary Materials

Supplementary figure S1 Induction of phosphorylated histone 2AX (γH2AX) by ataxia telangiectasia and Rad3-related (ATR) inhibition in bladder cancer cells showing high expression of apolipoprotein B mRNA editing enzyme catalytic subunit 3B (APOBEC3B). Findings for pChk1 and γH2AX. In HT1376 cells, an ATR inhibitor (VE-821) reduces pChk1 and induces γH2AX expression (evaluated by Western blotting), which is a marker of DNA damage.

Supplementary figure S2 Cell cycle analysis in **MIBC cells (a**. 5637 cells, b. HT-1197 cells).

## Data Availability

The data will not be shared because data related to human-derived materials may have ethical considerations and usage limitations.

## Supplementary Materials

The supplementary material is available online at https://doi.org/10.32604/or.2024.048919.
