# Supplementary figures and images for: New insights into ATR inhibition in muscle invasive bladder cancer: The role of apolipoprotein B mRNA editing catalytic subunit 3B
Source: Oncol Res. 2024 May 23;32(6):1021–30. doi: 10.32604/or.2024.048919 (PMC11136685; doi:10.32604/or.2024.048919)

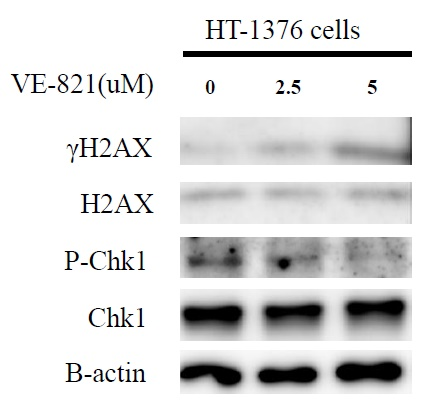

Supplement: Supplementary figure S1 [file OncolRes-32-48919-s001.tif]

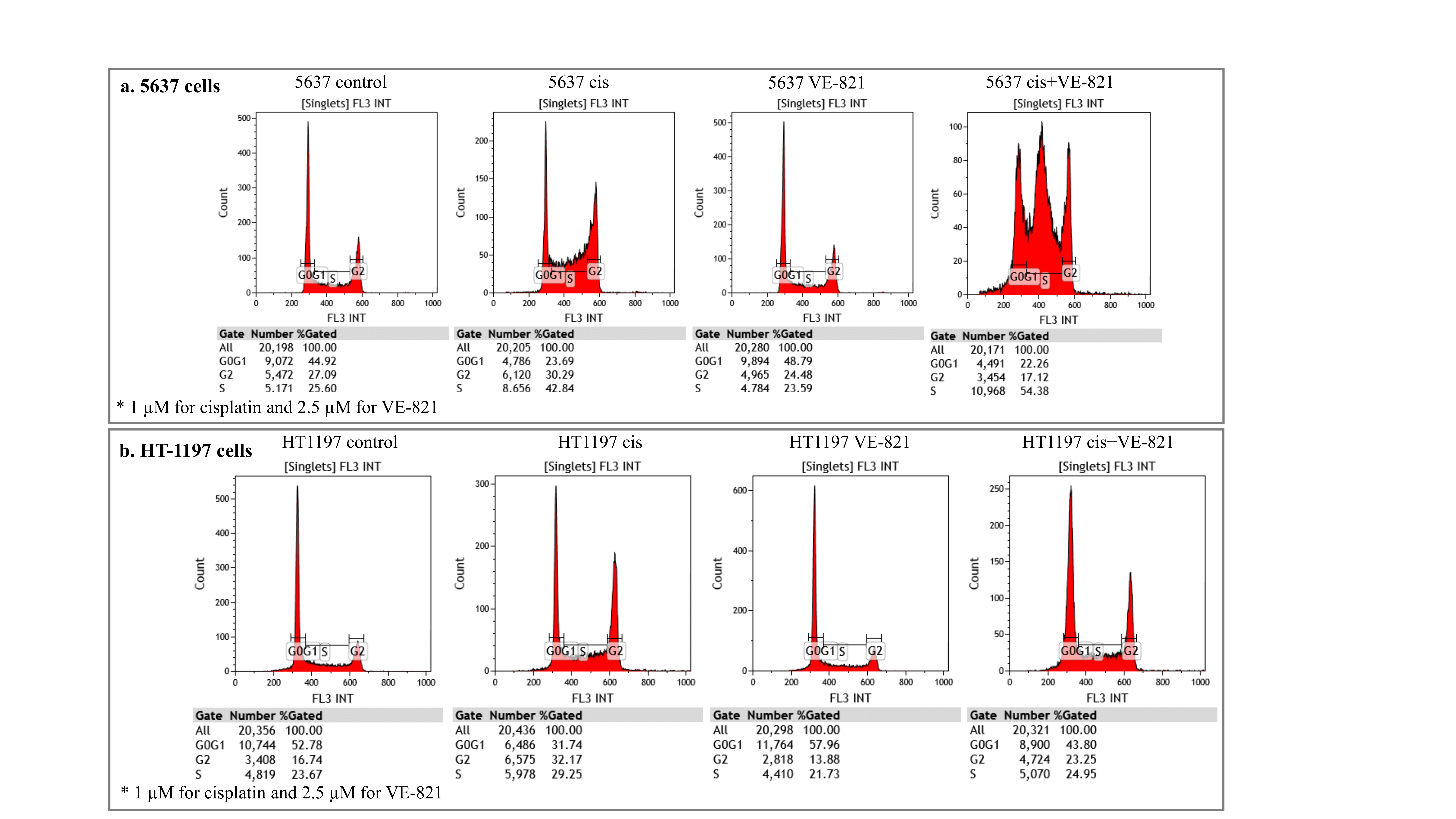

Supplement: Supplementary figure S2 [file OncolRes-32-48919-s002.tif]
