# Supplementary material for: New insights into ATR inhibition in muscle invasive bladder cancer: The role of apolipoprotein B mRNA editing catalytic subunit 3B
Source: Oncol Res. 2024 May 23;32(6):1021–30. doi: 10.32604/or.2024.048919 (PMC11136685; doi:10.32604/or.2024.048919)
Supplement: Supplementary file 3 [file OncolRes-32-48919-s001.docx]

**Supplementary table S1.** Baseline characteristics according to Apolipoprotein B mRNA editing enzyme catalytic subunit 3B (APOBEC3B) expression in patients with bladder cancer.

| **APOBEC3B expression** | | | | | |
| --- | --- | --- | --- | --- | --- |
|  |  | Total (n=61) | High (n=43) | Low (n=18) | *p-*value |
| **Age** | Range (median) | 51-86 (71) | 51-86 (72) | 61-84 (71) |  |
| **Sex** | Female | 11 (18%) | 7 (16%) | 4 (22%) | 0.717 |
|  | Male | 50 (82%) | 36 (84%) | 14 (78%) |  |
| **Primary site** | Bladder | 61 | 43 | 18 |  |
|  | Upper urinary tract | 0 | 0 | 0 |  |
| **T Stage** | 2 | 15 (25%) | 11 (26%) | 4 (22%) | 0.455 |
|  | 3 | 29 (48%) | 22 (51%) | 7 (39%) |  |
|  | 4 | 17 (27%) | 10 (23%) | 7 (39%) |  |
| **N stage** | negative | 40 (66%) | 29 (67%) | 11 (61%) | 0.635 |
|  | positive | 21 (34%) | 14 (33%) | 7 (39%) |  |
| **Stage** | II/III | 48 (79%) | 33 (77%) | 15 (83%) | 0.737 |
|  | IV | 13 (21%) | 10 (23%) | 3 (17%) |  |
| **Recurrence** | No | 20 (33%) | 13 (30%) | 7 (39%) | 0.363 |
|  | Yes | 23 (38%) | 15 (35%) | 8 (44%) |  |
|  | Not applicable | 18 (29%) | 15 (35%) | 3 (17%) |  |
| **Response** | No | 4 (7%) | 4 (9%) | 0 | 0.241 |
| **to cisplatina** | Yes | 20 (33%) | 16 37%) | 4 (22%) |  |
|  | Not applicable | 37 (60%) | 23 (54%) | 14 (78%) |  |
| **APOBEC3B** | negative | 0 | 0 | 0 |  |
| **expression** | 1+ | 2 (4%) | 0 | 2 |  |
|  | 2+ | 16 (26%) | 0 | 16 |  |
|  | 3+ | 43 (70%) | 43 | 0 |  |
|  | H-score, median | 210 | 234.7 | 133.9 | <0.0001 |

aTwenty-six patients were treated with first line palliative platinum- based chemotherapy. Among them, tumor response could not be evaluated in two patients because they refused further chemotherapy after one cycle, and follow-up discontinued. Moreover, 37 patients who were unsuitable for tumor response evaluation comprised 14 patients who received neoadjuvant or adjuvant chemotherapy and 21 patients who did not receive chemotherapy.
