# Supplementary material for: New insights into ATR inhibition in muscle invasive bladder cancer: The role of apolipoprotein B mRNA editing catalytic subunit 3B
Source: Oncol Res. 2024 May 23;32(6):1021–30. doi: 10.32604/or.2024.048919 (PMC11136685; doi:10.32604/or.2024.048919)
Supplement: Supplementary file 4 [file OncolRes-32-48919-s002.docx]

**Supplementary table S2.** Baseline characteristics according to and ataxia telangiectasia and Rad3-related (ATR) expression in patients with bladder cancer.

| **ATR expression** | | | | | |
| --- | --- | --- | --- | --- | --- |
|  |  | Total (n=61) | High (n=36) | Low (n=25) | *p*-value |
| **Age** | Range (median) | 51-86 (71) | 51-82 (67) | 59-86 (73) |  |
| **Sex** | Female | 11 (18%) | 9 (25%) | 2 (8%) | 0.106 |
|  | Male | 50 (82%) | 27 (75%) | 23 (92%) |  |
| **Primary site** | Bladder | 61 | 43 | 18 |  |
|  | Upper urinary tract | 0 | 0 | 0 |  |
| **T Stage** | 2 | 15 (25%) | 10 (28%) | 5 (20%) | 0.732 |
|  | 3 | 29 (48%) | 17 (47%) | 12 (48%) |  |
|  | 4 | 17 (27%) | 9 (25%) | 8 (32%) |  |
| **N stage** | negative | 40 (66%) | 24 (67%) | 16 (64%) | 0.829 |
|  | positive | 21 (34%) | 12 (33%) | 9 (36%) |  |
| **Stage** | II/III | 48 (79%) | 26 (72%) | 22 (88%) | 0.139 |
|  | IV | 13 (21%) | 10 (28%) | 3 (12%) |  |
| **Recurrence** | No | 20 (33%) | 13 (36%) | 7 (28%) | 0.036 |
|  | Yes | 23 (38%) | 9 (25%) | 14 (56%) |  |
|  | Not applicable | 18 (29%) | 14 (39%) | 4 (16%) |  |
| **Response** | No | 4 (7%) | 1 (3%) | 3 (12%) | 0.331 |
| **to cisplatina** | Yes | 20 (33%) | 13 (36%) | 7 (28%) |  |
|  | Not applicable | 37 (60%) | 22 (61%) | 15 (60%) |  |
| **ATR** | negative | 20 (33%) | 0 | 20 |  |
| **expression** | 1+ | 0 | 0 | 0 |  |
|  | 2+ | 5 (8%) | 0 | 5 |  |
|  | 3+ | 36 (58%) | 36 | 0 |  |
|  | H-score, median | 60 | 170 | 0 | <0.0001 |

aTwenty-six patients were treated with first line palliative platinum-based chemotherapy. Among them, tumor response could not be evaluated in two patients because they refused further chemotherapy after one cycle, and follow-up discontinued. Moreover, 37 patients who were unsuitable for tumor response evaluation comprised 14 patients who received neoadjuvant or adjuvant chemotherapy and 21 patients who did not receive chemotherapy.
